# Novel *MLH1* nonsense variant in a patient with suspected Lynch syndrome

**DOI:** 10.1038/s41439-024-00294-9

**Published:** 2024-09-17

**Authors:** Nobue Takaiso, Issei Imoto, Toshihiko Matsumoto, Akiyo Yoshimura

**Affiliations:** 1 Risk Assessment Unit, Aichi Cancer Center Hospital, Nagoya, Japan; 2 Aichi Cancer Center Research Institute, Nagoya, Japan; 3 Department of Medical Oncology, Ichinomiyanishi Hospital, Ichinomiya, Japan; 4 Department of Breast Oncology, Aichi Cancer Center Hospital, Nagoya, Japan

**Keywords:** Genetic testing, Cancer genetics

## Abstract

Loss-of-function germline variants of *MLH1* cause Lynch syndrome. Here, we present the case of a 43-year-old male patient diagnosed with cecal and transverse colon adenocarcinomas. The characteristics of the case met the revised Bethesda guidelines, and the tumors demonstrated a high frequency of microsatellite instability. Genetic testing for mismatch repair genes (indicative of Lynch syndrome) revealed a novel heterozygous germline pathogenic variant, NM_000249.4:c.856A>T/NP_000240.1:p.(Lys286Ter), in *MLH1*.

Lynch syndrome (LS, OMIM#120435) is an autosomal dominant cancer predisposition syndrome that accounts for approximately 1–3% of all colorectal cancers (CRCs) and is associated with an increased risk of extracolonic malignancies, such as endometrial, ovarian, stomach, small bowel, hepatobiliary, and urothelial cancers^1^. LS is caused either by germline loss-of-function (LoF) pathogenic (P) and likely pathogenic (LP) variants in one of the four DNA mismatch repair (MMR) genes (*MLH1*, *MSH2*, *MSH6*, and *PMS2*) or by germline deletions in the epithelial cell adhesion molecule (*EPCAM*) gene leading to epigenetic silencing of the adjacent *MSH2*^1,2^. The microsatellite instability (MSI) phenotype is a hallmark of LS-associated tumors caused by MMR system deficiency (dMMR)^2^. For the definitive diagnosis of LS, genetic testing of these MMR genes is currently used in clinical practice. Therefore, accumulated knowledge regarding germline variants in MMR genes is necessary for the accurate diagnosis of LS. Genetic identification of LS patients not only alerts the probands to their own life and health risks but also warns their relatives of their own cancer risk and enables subsequent genetic testing, with significant benefits in terms of the timing, cost, and effectiveness of surveillance, early detection, and reduced cancer mortality.

*MLH1* and *MSH2* are the major pathogenic genes for LS^2^. Additionally, the majority of variants of *MLH1* and *MSH2* reported in one of the disease-related databases (InSiGHT variant database, https://www.insight-group.org/variants/databases/) are truncated (predominantly nonsense or frameshift variants)^2^, frequently leading to the LoF of these genes. Here, we report a novel *MLH1* nonsense variant, NM_000249.4:c.856A>T/NP_000240.1:p.(Lys286Ter), associated with LS and classified as LP according to the joint consensus guidelines of the American College of Medical Genetics and Genomics and the Association for Molecular Pathology (ACMG/AMP)^3^.

A 43-year-old Japanese male (III-2, Fig. 1) was admitted with sudden abdominal pain. The patient had no significant medical history. Through imaging evaluation via abdominal computed tomography (CT), the patient was diagnosed with perforation of the cecal and transverse colon and panperitonitis. On the same day, the patient underwent an emergency right hemicolectomy. Pathological examination revealed pT3N3 adenocarcinoma on the basis of the TNM classification^4^ for both cecal and transverse colon cancers. Detailed imaging examinations via contrast-enhanced CT and magnetic resonance imaging before postoperative chemotherapy revealed multiple metastases in the liver. The results of MSI testing of the resected CRC tumors demonstrated a high frequency of MSI (MSI-H); however, the tumors were negative for the BRAF V600E variant.

**Fig. 1:**
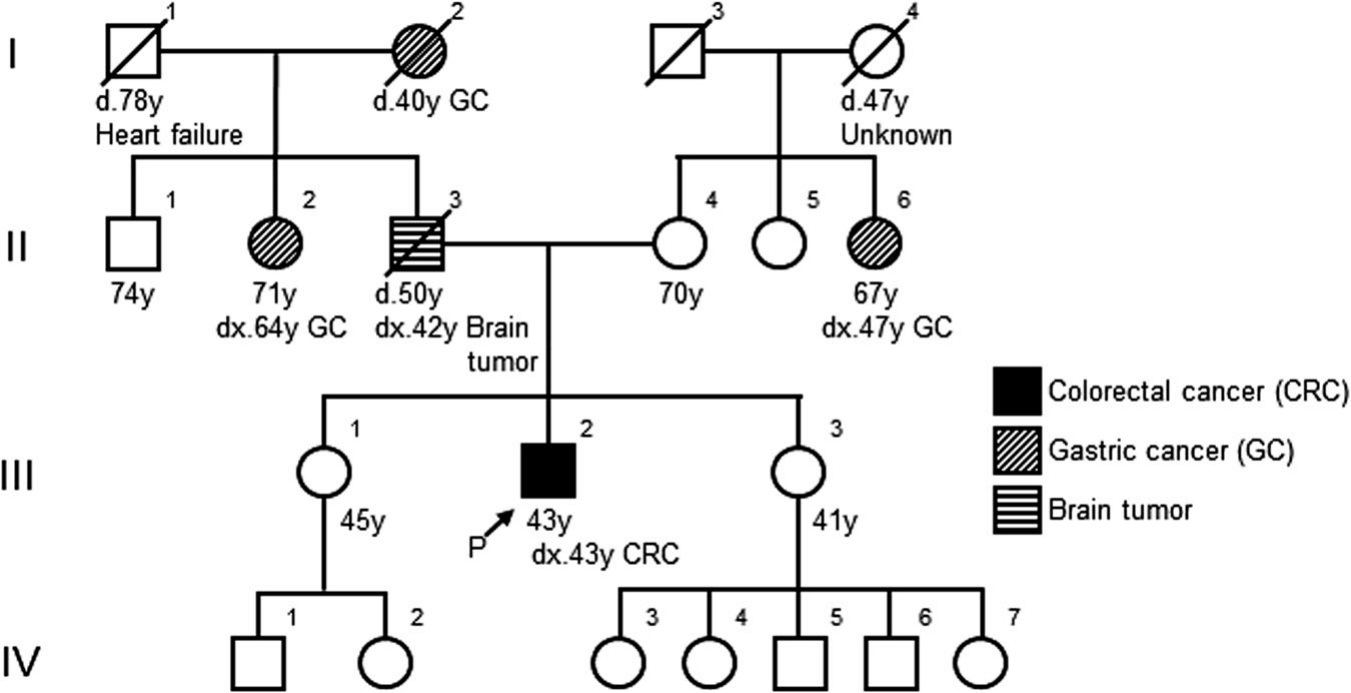
Family pedigree. The arrow indicates the proband (P).

Additionally, the characteristics of the case met the criteria of the revised Bethesda guidelines^5^; therefore, the patient was referred to the Clinical Genetics Department of our hospital for hereditary tumor risk assessment, although the characteristics of the family members did not meet the Amsterdam Criteria II for LS^6^ (Fig. 1). Several commercially available genetic tests have been proposed by clinical geneticists for the definitive diagnosis of hereditary CRC. Following pretest genetic counseling and the acquisition of informed consent, the patient opted for and underwent MMR gene testing for the evaluation of the *MLH1*, *MSH2*, *MSH6*, *PMS2*, and *EPCAM* genes via very long amplicon sequencing (vLAS), an optimized long-range polymerase chain reaction (PCR)-based next-generation sequencing method (Center for Clinical Genomics, Kanazawa Medical University Hospital, Uchinada, Japan)^7^. Using vLAS technology, single-nucleotide variants (SNVs), small insertions or deletions (indels), large indels, and structural variants, including exon-level copy number variants (CNVs) within the regions covered by long-range PCR, can be detected. The identified variants were interpreted on the basis of ACMG/AMP Guidelines^3^.

The heterozygous nonsense variant NM_000249.4:c.856A>T/NP_000240.1:p.(Lys286Ter) (NC_000003.12:g.37017571A>T) in *MLH1* was identified and confirmed via Sanger sequencing (Fig. 2). To our knowledge (4 June 2024 date last accessed), this SNV has never been reported in disease-related databases, including the Human Gene Mutation Database (HGMD) Professional (https://my.qiagendigitalinsights.com/bbp/view/hgmd/pro/start.php), Leiden Open Variation Database (LOVD) v3.0 (https://www.lovd.nl/), InSiGHT variant database, or ClinVar (https://www.ncbi.nlm.nih.gov/clinvar/), and has rarely been reported in population databases (PM2 ACMG/AMP variant criterion^3^), including gnomAD v4.1.0 (https://gnomad.broadinstitute.org/, allele frequency = 6.842 × e^−7^) and 54KJPN-SNV/INDEL (https://jmorp.megabank.tohoku.ac.jp/, allele frequency = 0). This SNV is predicted to generate a stop codon, possibly leading to a premature termination codon and causing a LoF (PVS1 ACMG/AMP variant criterion^3^) via nonsense-mediated mRNA decay (NMD). According to the ACMG/AMP guidelines^3^, this SNV was classified as LP on the basis of the PM2 and PVS1 criteria. No other variants that could be responsible for LS were detected in any of the tested genes. Therefore, the patient was diagnosed with LS due to a novel germline nonsense variant of *MLH1*. Following the diagnosis of the proband, *MLH1* genetic testing of at-risk family members, especially unaffected first-degree relatives, was suggested for genetic counseling but has not yet been performed.

**Fig. 2:**
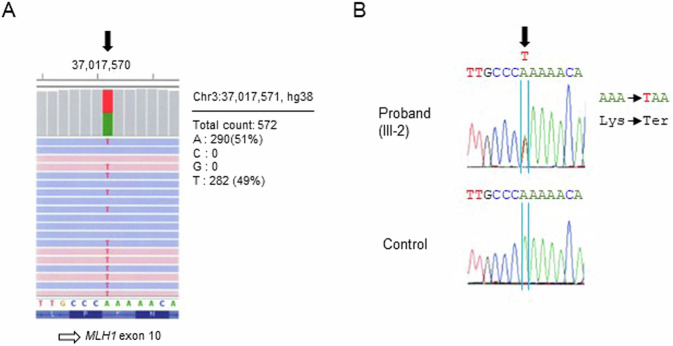
Novel heterozygous nonsense variant of *MLH1*. **A** Integrative Genomics Viewer (IGV) snapshot of NM_000249.4:c.856A>T (NC_000003.12:g.37017571A>T, filled arrow). **B** Sanger sequencing confirmation of NM_000249.4:c.856A>T (filled arrow) in *MLH1*. Direct PCR sequencing analysis was performed using genomic DNA from a patient peripheral blood sample (III-2 in Fig. 1) and a control sample.

At the same nucleotide position, NM_000249.4:c.856, two other nucleotide changes, A > C and A > G, which cause missense substitutions of amino acids at codon 286, p.(Lys286Gln) and p.(Lys286Glu), respectively, have been reported in disease-related databases as variants of uncertain significance or as benign or likely benign variants. Because nonsense variants in neighboring codons, such as codon 284, have been reported as P or LP in the ClinVar and InSiGHT databases, it is reasonable to predict that c.856A>T is a null variant causing NMD to lead to LS, although the nonsense SNV in codon 286 has not been reported previously. Identifying a pathogenic MMR variant in the proband is essential to confirm the genetic predisposition to LS in the proband and enable the presymptomatic diagnosis of variant carriers in family members. Therefore, reporting novel pathogenic variants responsible for LS will help in the accurate diagnosis of LS.

## HGV datbase

The relevant data from this Data Report are hosted at the Human Genome Variation Database at 10.6084/m9.figshare.hgv.3439.

## Supplementary information

COI disclosure
